# Alterations of the gut microbiome in Chinese patients with systemic lupus erythematosus

**DOI:** 10.1186/s13099-016-0146-9

**Published:** 2016-12-08

**Authors:** Zhixing He, Tiejuan Shao, Haichang Li, Zhijun Xie, Chengping Wen

**Affiliations:** College of Basic Medical Science, Zhejiang Chinese Medical University, Hangzhou, 310053 China

**Keywords:** Gut microbiome, Systemic lupus erythematosus, Chinese patients, Illumina Miseq, Genus level

## Abstract

**Background:**

Systemic lupus erythematosus (SLE) in patients from Spain is associated with intestinal dysbiosis. This study explores whether the alteration of the gut microbiome in SLE patients from China is consistent with the intestinal dysbiosis of SLE patients from Spain.

**Results:**

The depletion of Firmicutes and the enrichment of Bacteroidetes in SLE patients from China were consistent with the SLE patients from Spain. Furthermore, we found that nine genera of gut microbiota were SLE-related microorganisms in Chinese subjects. Genera *Rhodococcus*, *Eggerthella*, *Klebsiella*, *Prevotella*, *Eubacterium, Flavonifractor* and *Incertae sedis* were significantly enriched, while genera *Dialister* and *Pseudobutyrivibrio* were significantly depleted in SLE patients. Receiver operating characteristic analysis indicated that the nine genera have the potential to distinguish SLE patients from healthy controls.

**Conclusions:**

Comparing the dysbiosis of the gut microbiome among SLE patients from China or Spain, may indicate that the gut microbiome profiles of SLE patients are more influenced by disease than ethnicity.

## Background

The human gut harbors diverse and abundant microbes that benefit us by training the immune system [1], protecting against opportunistic pathogens [2], and harvesting nutrients and energy [3]. Recently, increased evidence indicates that intestinal dysbiosis is associated with human diseases, such as cancer [4], liver cirrhosis [5], and autoimmune diseases [6, 7]. Systemic lupus erythematosus (SLE), a complex autoimmune disease characterized by its higher frequency in women, is associated with the gut microbiome. Compared to healthy controls, the SLE patients from Spain were characterized by increased Bacteroidetes levels and a lower Firmicutes/Bacteroidetes ratio [8]. In addition, the species *Lactobacillus* was applied in management of SLE [9]. Therefore, the gut microbiome can serve as new biomarkers or therapy for SLE.

It is well known that the human gut microbiome could be susceptible to the host’s genotype [10], age [11], sex [12] and diet [13]. The association of the gut microbiome with diseases may be diverse due to the above factors. For example, the data of the association between the gut microbiome and obesity varied in different ethnicities [14–16]. The reason may be that pronounced differences in the gut microbiome existed among different ethnicities [11]. China and Spain belong to two different continents with dissimilar genes and diets. Therefore, the alterations of the gut microbiome associated with SLE should be variable in SLE patients from China versus Spain.

In this study, Illumina Miseq sequencing was employed to investigate the alterations of the gut microbiome in SLE patients from China. The overall goal of the study was to ascertain whether the gut microbiome alteration of SLE in a Chinese population could be used as a biomarker and to evaluate differences compared to SLE biomarkers from Spain.

## Methods

### Study subjects

We performed a cross-sectional collection of fecal samples from female patients diagnosed with SLE from three hospitals (Zhejiang Provincial Hospital of TCM, the Second Affiliated Hospital of Zhejiang Chinese Medical University, and Zhejiang Province People Hospital). The diagnosis of SLE was made according to the criteria set by the American College of Rheumatology (ACR) [17, 18]. After the diagnosis, the recruited patients suffered from SLE for at least 1 year, did not receive any medical treatment with 1 month of study participation, and had no comorbid disorders. Forty-five female patients were included in this study, and their clinical diagnosis and blood examination reports were obtained from the hospitals. Forty-eight female volunteers were recruited by a routine physical examination. The healthy controls had no gastrointestinal tract disorders and did not receive antibiotics within 1 month of this study. In addition, there were no significant differences among the two groups in terms of age, smoking history, and alcohol or dietary intake. All subjects included in this study provided written informed consent, and the protocol of this study was approved by the Ethics Committee of Zhejiang Chinese Medical University. Clinical data such as body mass index (BMI), erythrocyte sedimentation rate (ESR), SLE disease activity index (SLEDAI) and disease duration are shown in Table 1.

**Table 1:** Demographic and clinical chemistry characteristics of human subjects

Characteristics	SLE patients		Healthy controls	
	Train	Test	Train	Test
Sample numbers	35	10	35	13
Age mean ± SD [min, max]	46.0 ± 1.8 [25, 61]	39.9 ± 4.3 [18, 62]	43.5 ± 2.4 [22, 68]	42.7 ± 1.9 [20, 56]
BMI (kg/m^2^) mean ± SD [min, max]	21.5 ± 0.6 [16.4, 28.8]	21.2 ± 1.2 [18.3, 27.7]	22.1 ± 1.0 [20.5, 27.7]	21.6 ± 0.2 [20.6, 25.8]
ESR (mm/h) ± SD [min, max]	14.8 ± 3.3 [3, 38]	14.6 ± 3.9 [8, 46]	6.9 ± 0.6 [4, 15]	7.2 ± 0.4 [3, 12]
SLEDAI ± SD [min, max]	7.5 ± 0.5 [3, 14]	6.7 ± 0.8 [4, 10]	–	–
Disease duration (years) ± SD [min, max]	7.9 ± 1.2 [1, 28]	5.0 ± 1.6 [1, 16]	–	–

### Illumina Miseq sequencing of 16S rRNA gene-based amplicons and data processing

Total DNA was extracted from thawed fecal samples using the QIAamp^®^ Fast DNA Stool Mini Kit (Qiagen, Hilden, Germany) according to the manufacturer protocols. The V3–V4 regions of the bacterial 16S rRNA gene sequences were amplified from the diluted DNA extracts with the primers 319F (5′-ACTCCTACGG GAGGCAGCAG-3′) and 806R (5′-GGACTACHVGGGTWTCTAAT-3′). PCR amplification was performed in a 30 μl mixture containing 0.5 μl of DMSO, 1.0 μl of forward primer (10 mM), 1.0 μl of reverse primer (10 mM), 5.0 μl of DNA sample, 7.5 μl of ddH_2_O and 15.0 μl of Phusion High-Fidelity PCR Master Mix with HF Buffer (NEB). The reactions were hot-started at 98 °C for 30 s, followed by 30 cycles of 98 °C for 15 s, 58 °C for 15 s, and 72 °C for 15 s, with a final extension step at 72 °C for 1 min. PCR products were purified using a QIAquick Gel Extraction kit (Qiagen, Valencia, CA, USA). The amplicon library was prepared using a TruSeq™ DNA sample preparation kit (Illumina Inc, San Diego, CA). The sequencing reaction was conducted using Illumina MiSeq platforms (Illumina Inc, San Diego, CA).

After sequencing, the data were analyzed on the Quantitative Insights Into Microbial Ecology (QIIME, www.qiime.org) platform using the default parameters [19]. Before assembly, sequence reads were first filtered to remove low-quality or ambiguous reads, including reads lacking exact matching with the primer, reads containing ambiguous character (N), and reads with an average quality score <25. Only two reads with a sequence overlap longer than 20 bp were assembled. The assembled sequence reads with <400 bp or >500 bp were discarded. High-quality sequences were binned into 16S rRNA Operational Taxonomic Units (OTUs) and defined at ≥97% sequence homology. Chimera detection and removal were assessed using the GOLD reference databased and uchime [20]. Taxonomic affiliation of each OTU was performed with QIIME against the SILVA database [21]. Alpha diversity (PD_whole_tree, observed species, Simpson, Shannon, Singles, Doubles) and beta diversity (unweighted UniFrac) were performed in QIIME to 1000 reads, according to the OTU table rarefied and 20,000 reads were finally extracted from each sample for other analyses.

### Statistical analysis

To test whether gut microbial species could be differentiated between SLE patients and healthy controls, a metric multidimensional scaling method based on projection known as principal coordinates analysis (PCoA) was used. Each sample was mapped based on the overall microbial composition and assessed for similarities.

Two datasets were created from all study subjects to investigate whether microbial biomarkers were available for analysis. The first dataset (train) was used to create the diagnostic algorithm. The above algorithm was then applied to the second dataset (test) for the purpose of evaluating how accurately microbial biomarkers were identified. All study subjects were randomly and independently divided into train or test datasets.

To visualize the potential of significantly altered genera as SLE biomarkers, receiver operating characteristic (ROC) curves were plotted in the statistical programming language R (V.3.1.2). ROC curves are an effective method of evaluating the quality or performance of diagnostic tests and are widely used in gut microbiomes to evaluate the performance of many microbial biomarkers. Area under the curves (AUCs) of ROC was calculated to evaluate the performance of the fitted logistic regression models. The AUCs were based on the predicted probability of SLE for each individual, using the multivariate logistic regression coefficient estimates together with the individual’s transformed relative abundances for each bacterial taxon included in the analysis.

Microbial phylum and genera concentrations between SLE patients and healthy controls were compared using a Mann–Whitney non-parametric test in SPSS software 16.0.

## Results

### Structure and composition of the gut microbiome in SLE

**Fig. 1:**
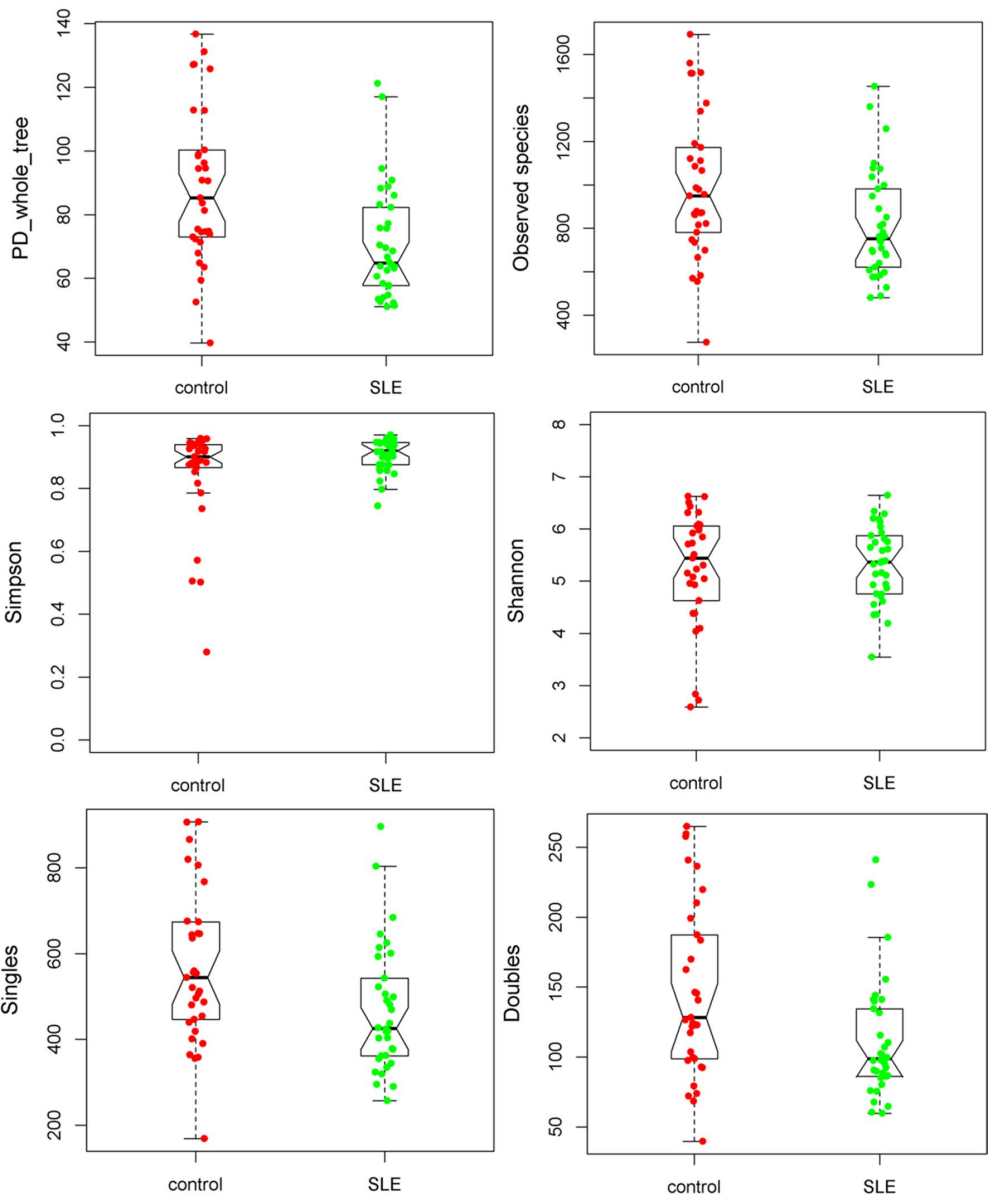
The alpha-diversity indices between healthy controls and SLE patients from China

**Fig. 2:**
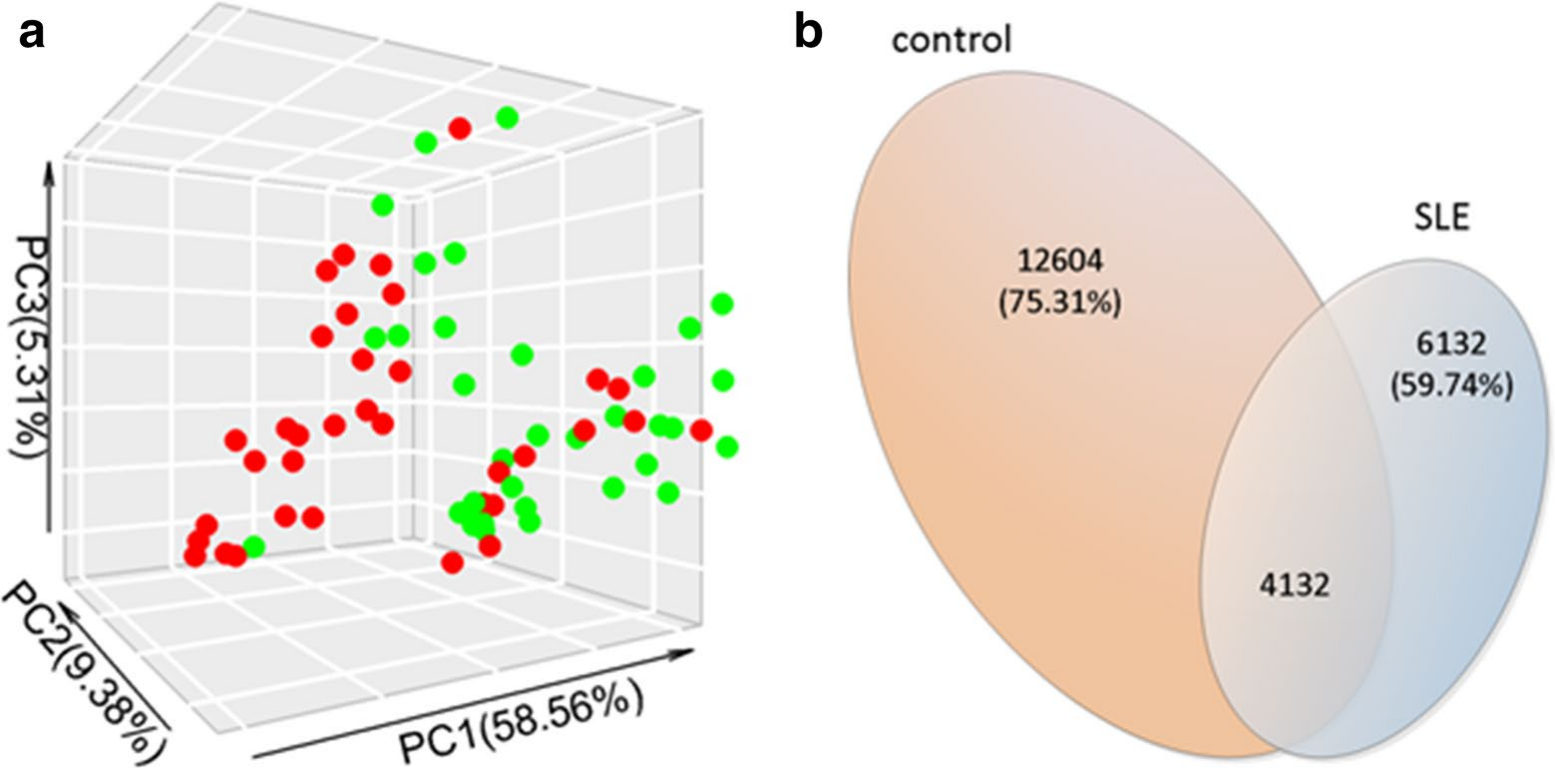
**a** PCoA score plots of SLE patients (*green*) and healthy controls (*red*) based on the gut microbial composition. **b** Venn diagrams show the percentage of the shared OTUs between SLE patients and healthy controls

To assess overall differences in microbial community structure in SLE patients and healthy controls, we calculated the measures of alpha and beta diversity, representing microbial diversity in each sample and differences among samples, respectively. As shown in Fig. 1, not all of the alpha-diversity indices were different. PD_whole_tree, observed species, Singles and Doubles were significantly higher in healthy controls than in SLE patients, but there was no difference in Shannon and Simpson. The above results indicate that lower diversity, richness and rare OTUs of gut microbiome existed in SLE patients, but the evenness of the gut microbiome could not differentiate SLE patients from healthy controls. The beta-diversity difference using the weighted UniFrac distance was shown in PCoA analysis (Fig. 2a). The ordination plot demonstrated a clear difference between SLE patients and healthy controls. Examining shared OTUs showed that a total of 4132 OTUs were shared between SLE patients and healthy controls, which accounted for 24.69% of the total OTUs in healthy controls and 40.26% of the total OTUs in SLE patients respectively (Fig. 2b).

### SLE-associated microbiota changes

**Fig. 3:**
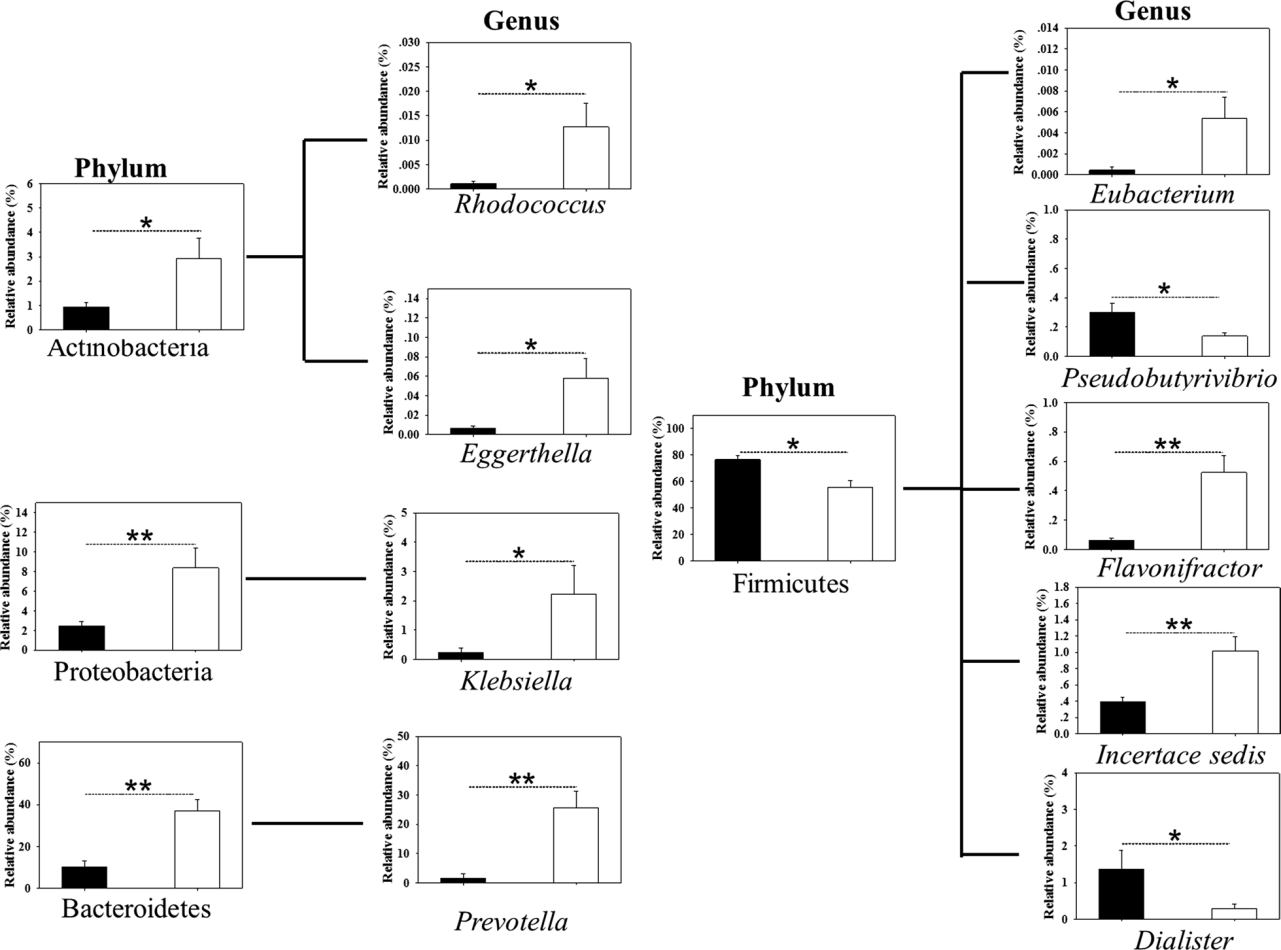
Significantly altered gut microbiota between SLE patients and healthy controls at the phylum and genus levels. “**” denotes *p* < 0.01, “*” denotes *p* < 0.05. The *black column* denotes healthy controls, and the *blank column* denotes SLE patients

At the phylum level, the gut microbiota of both groups was dominated by Firmicutes and Bacteroidetes, which contributed to 99% of the gut microbiota, with smaller contributions of Proteobacteria, Actinobacteria and Fusobacteria. The significantly altered phylum between SLE patients and healthy controls is shown in Fig. 3. Compared to healthy controls, SLE patients had significantly increased relative abundances of the phylum Bacteroidetes, Actinobacteria and Proteobacteria and a decreased relative abundance of the phylum Firmicutes.

We next investigated whether the relative abundances of the gut microbiome differed between SLE patients and healthy controls at the genus level (Fig. 3). In the phylum Bacteroidetes, only the levels of the genera *Prevotella* were higher in SLE patients than in healthy controls. Five significantly altered levels of genera were found in phylum Firmicutes; genera *Pseudobutyrivibrio* and *Dialister* decreased in SLE patients, while *Eubacterium*, *Flavonifractor*, and *Incertae sedis* increased in SLE patients. Genera *Klebsiella* of the phylum Proteobacteria was found in lower amounts in healthy controls than in SLE patients. Genera *Rhodococcus* and *Eggerthella* of the phylum Actinobacteria were more abundant in SLE patients.

### Evaluation of the performance of significantly changed genera

**Fig. 4:**
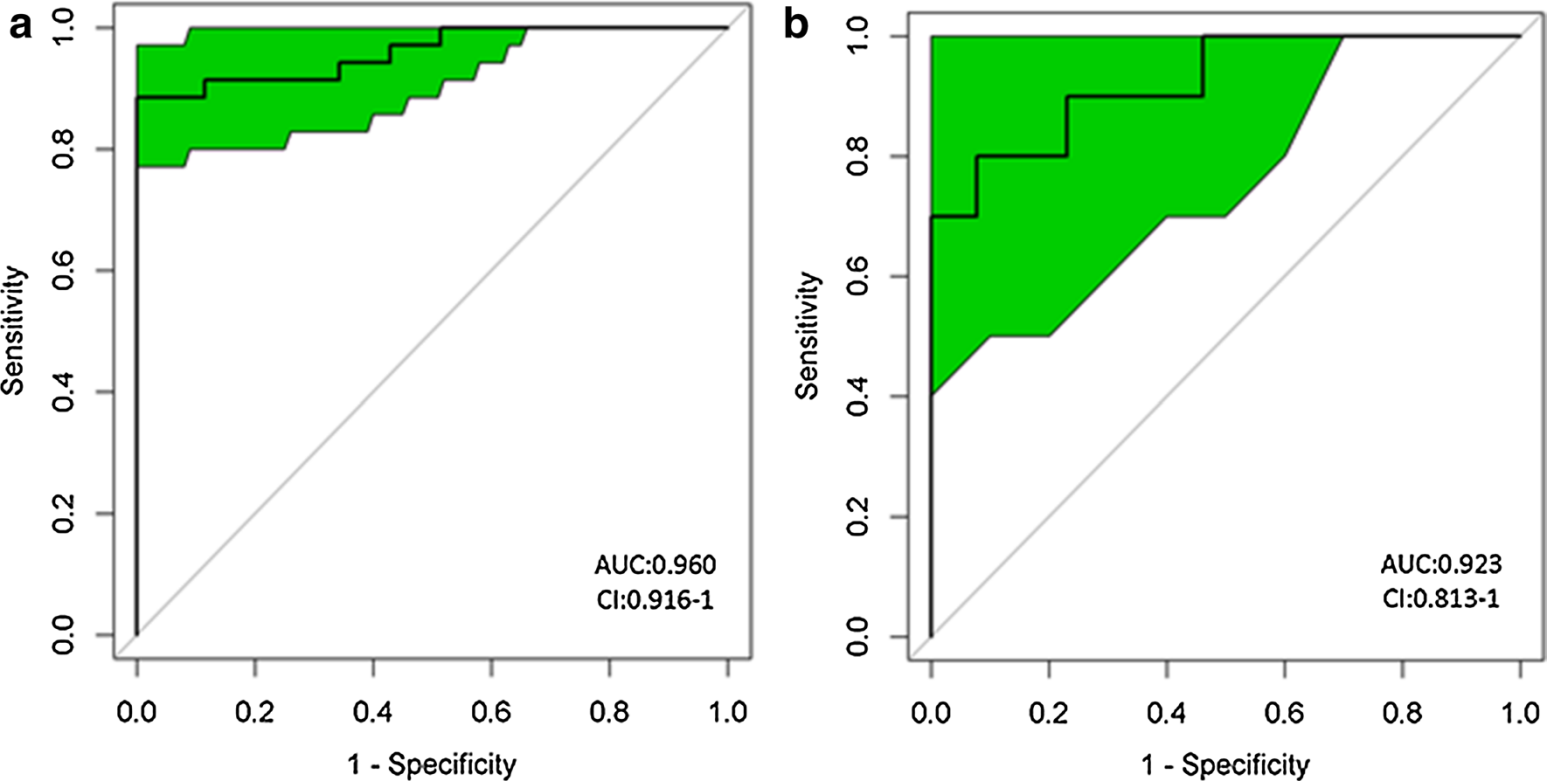
Receiver operating characteristic (ROC) curves demonstrating the performance of significantly altered microbial genera for train (**a**) and test (**b**) validation subjects using leave-one-out cross-validation

To evaluate the utility of the differentially abundant genera as potential biomarkers, we conducted a ROC analysis. Nine significantly altered genera were screened for their abilities in distinguishing SLE patients and healthy controls. Complete results of train and test validation subjects using leave-one-out cross-validation are shown in Fig. 4. The combined significantly altered genera have the ability to differentiate between SLE patients and healthy controls with AUC = 0.960, 95% Cl 0.916–1.0 in the train validation subjects and AUC = 0.923, 95% Cl 0.813–1.0 in the test validation subjects.

## Discussion

The clinical and immunological abnormalities of SLE among different ethnicities were known to widely vary because of diverse pathogenesis (genetic, environmental, hormonal, etc.,) [22]. Therefore, the alterations of gut microbiome associated with SLE should display differences among separate ethnicities.

Firstly, a significant depletion of Firmicutes and enrichment of Bacteroidetes in SLE patients were found both in Spanish and Chinese subjects. Phylum Firmicutes and Bacteroidetes account for more than 90% of all phylogenetic species and are involved in host metabolism and immunity [1, 23]. Previous studies have described that the depletion of Firmicutes and the enrichment of Bacteroidetes in the human gut microbiota were associated with some disorders [8]. Other studies also showed that an altered balance between Bacteroidetes and Firmicutes was correlated with clinical states [24]. Therefore, the influence of SLE on human gut microbiome at the phylum level was not characterized by ethnicity. Therefore, the disorders of SLE are strongly related to the depletion of Firmicutes and the enrichment of Bacteroidetes in Spanish and Chinese patients.

Secondly, certain differences in gut microbiome were found among SLE patients from Spain and China. At the family level, SLE patients from Spain showed a depletion of *Lachnospiraceae* and *Ruminococcaceae* and an enrichment of *Bacteroidaceae* and *Prevotellaceae* [8]. Our study indicated that only the family *Prevotellaceae* showed significant increases in SLE patients versus healthy controls from China (data not shown). However, this study employed Illumina Miseq sequencing to uncover some differences at the genus level between SLE patients and healthy controls from China. Significantly altered genera in SLE patients of China were mostly related to the intestinal disturbance of autoimmune disorders. Genus *Rhodococcus* infection was observed with Crohn’s disease [25]. Genera *Eggerthella, Klebsiella*, *Prevotella* and *Dialister* were strongly positive for rheumatoid arthritis or ankylosing spondylitis [26–29]. Genus *Pseudobutyrivibrio*, reduced in SLE patients, displayed decreased levels in the gut microbiota profile of psoriatic arthritis [30]. Genus *Eubacterium* was enriched in SLE patients of China, but decreased in multiple sclerosis patients [31]. The disorders of the gut microbiome at the genera level in SLE patients from China were similar to the gut microbiome profiles of other autoimmune diseases. Thus, a combination of these genera could distinguish SLE patients from healthy controls.

As mentioned above, similarities at the phylum level and differences at family and genus levels of SLE-related gut microbiota existed between Spanish and Chinese subjects. The sensitivity of different taxon levels in evaluating microbial community may result in the above phenomenon. The gut microbiota appears to impose a greater influence on the detailed taxon of microorganisms. Therefore, the correlation of SLE with gut microbiota at the phylum level was more stable than that at the family or genera level. However, the differences of SLE-related gut microbiota at family and genera levels between Spanish and Chinese populations may be attributed to the differences of the host’s genetics and diet among the two ethnic groups.

## Conclusions

Alterations of the gut microbiome in SLE patients from China and Spain were shown to be consistent at the phylum level with little differences at the family level. However, our study further revealed an alteration of the gut microbiome in SLE patients at the genus level. Significantly altered genera could thus have the ability to discriminate between SLE patients and healthy controls.
